# Mito-phylogenetic relationship of the new subspecies of gentle monkey *Cercopithecus mitis manyaraensis*, Butynski & De Jong, 2020

**DOI:** 10.5194/pb-9-11-2022

**Published:** 2022-06-29

**Authors:** Dietmar Zinner, Sascha Knauf, Idrissa S. Chuma, Thomas M. Butynski, Yvonne A. De Jong, Julius D. Keyyu, Rehema Kaitila, Christian Roos

**Affiliations:** 1 Cognitive Ethology Laboratory, Deutsches Primatenzentrum, Leibniz Institute for Primate Research, 37077 Göttingen, Germany; 2 Department of Primate Cognition, Georg-August-University of Göttingen, 37077, Göttingen, Germany; 3 Leibniz Science Campus Primate Cognition, 37077 Göttingen, Germany; 4 Work Group Neglected Tropical Diseases, Infection Biology Unit, Deutsches Primatenzentrum, 37077 Göttingen, Germany; 5 Institute of International Animal Health/One Health, Friedrich-Loeffler-Institut, Federal Research Institute for Animal Health, 17493 Greifswald, Insel Riems, Germany; 6 Tanzania National Parks (Serengeti National Park), Arusha, Tanzania; 7 Eastern Africa Primate Diversity and Conservation Program & Lolldaiga Hills Research Programme, Nanyuki, Kenya; 8 Tanzania Wildlife Research Institute, Arusha, Tanzania; 9 Tanzania National Parks (Lake Manyara National Park), Arusha, Tanzania; 10 Primate Genetics Laboratory, Deutsches Primatenzentrum, Leibniz Institute for Primate Research, 37077 Göttingen, Germany; 11 Gene Bank of Primates, Deutsches Primatenzentrum, Leibniz Institute for Primate Research, 37077 Göttingen, Germany

## Abstract

In 2020, a new subspecies was described in the *Cercopithecus mitis* complex, the Manyara monkey *C. m. manyaraensis*, Butynski & De Jong, 2020. The internal taxonomy of this species complex is still debated, and the phylogenetic relationships among the taxa are unclear. Here we provide the first mitochondrial sequence data for *C. m. manyaraensis* to determine its position within the mitochondrial phylogeny of *C. mitis*. This subspecies clusters within the youngest (internal divergences between 1.01 and 0.42 Ma) of three main taxonomic clades of *C. mitis*. Its sister lineages are *C. m. boutourlinii* (Ethiopia), *C. m. albotorquatus* (Kenya and Somalia), *C. m. albogularis* (Kenya and Tanzania), and *C. m. monoides* (Tanzania and Mozambique). In general, the phylogenetic tree of *C. mitis* based on mitochondrial sequence data indicates several paraphyletic relationships within the *C. mitis* complex. As in other African cercopithecines (e.g. *Papio* and *Chlorocebus*), these data are suitable for reconstructing historic biogeographical patterns, but they are only of limited value for delimitating taxa.

## 1 Introduction

**Figure 1:**
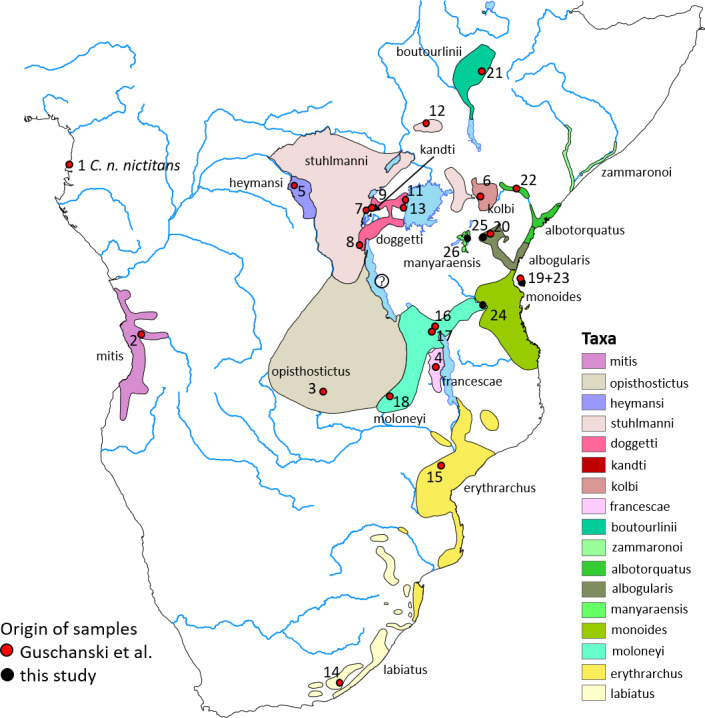
Approximate distribution of gentle monkey *Cercopithecus mitis* taxa based on the IUCN range map (updated from Butynski and de Jong, 2019, 2020). *Cercopithecus m. francescae* is no longer recognized by IUCN and is considered a synonym of *C. m. moloneyi*. The taxonomic status of *C. mitis* in the Mahale Mountains, central western Tanzania, is unclear (indicated by a question mark). Numbers refer to genetic samples from Guschanski et al. (2013) (red dots) or this study (black dots). The geographic origin of sample 10 is unclear (zoo animal) and is, therefore, not depicted on this map. See Table S2. Basemap by ESRI.

The gentle monkey *Cercopithecus mitis* Wolf, 1822 is an arboreal primate that is widely distributed in the forests of central, eastern, and south-eastern Africa (Fig. 1; Kingdon, 1971, 2013; Butynski, 1990; Lawes, 1990; Lawes et al., 2013). This species is highly polytypic, and its taxonomy is complex and extensively debated (Hill, 1966; Dandelot, 1974; Napier, 1981; Groves, 2001; Grubb, 2001; Grubb et al., 2003; Kingdon, 2013, 2015; Lawes et al., 2013; Dalton et al., 2015; Butynski and de Jong, 2020; Table S1 in the Supplement). At present, 16 subspecies are recognized by the International Union for Conservation of Nature (IUCN), under which no fewer than 28 major synonyms have been placed (Lawes et al., 2013). The subspecies are predominantly founded on phenotypic traits (colouration and pattern of the pelage) and geographic distributions (Butynski and de Jong, 2020). Here we apply the taxonomic arrangement for *C. mitis* as adopted at the IUCN SSC African Primate Red List Assessment Workshop in Rome in April 2016 but also recognize *C. m. francescae* (Butynski and De Jong, 2019).

The most recently described subspecies of gentle monkeys is the Manyara monkey *C. m. manyaraensis*, Butynski & De Jong, 2020. This subspecies is endemic to the forests of Lake Manyara National Park (NP), Ngorongoro Conservation Area, and up to about 40 km south of Lake Manyara, central northern Tanzania. Booth (1968) considered *C. mitis* in this region to represent a hybrid swarm between the geographically two closest subspecies, *C. m. stuhlmanni* and *C. m. albogularis* (Fig. 1). Butynski and de Jong (2020) described *C. m. manyaraensis* based on its geographical isolation (the nearest *C. mitis* subspecies is separated by > 90 km of unsuitable semi-arid habitat), distinct colouration and pattern of the pelage, and little intra-population variation. The known geographic range is about 1480 km ${}^{2}$ , the expected geographic range is roughly 5865 km ${}^{2}$ , and the known altitudinal range is 960 m a.s.l. to at least 2550 m a.s.l.

Few molecular studies that included *C. mitis* have been conducted. These studies, based mainly on mitochondrial markers, were not able to clarify the phylogenetic relationships among the various *C. mitis* taxa. They did, however, suggest rapid range expansion and radiation (Turner et al., 1988; Guschanski et al., 2013; Dalton et al., 2015; Larkin-Gero 2020). Butynski and de Jong (2020) expressed the need for more molecular studies to further understand the evolutionary history and taxonomy of *C. mitis*, in particular the phylogenetic position of *C. m. manyaraensis.* Here we provide the first mitochondrial sequence data for *C. m. manyaraensis* and determine its position within the mitochondrial phylogeny of *C. mitis*.

## 2 Materials and methods

### 2.1 Genetic samples

Blood samples from *C. mitis* were collected in Lake Manyara NP ( n = 4), Arusha NP ( n = 2), Udzungwa Mountains NP ( n = 4), and on Zanzibar Island ( n = 1) during a Tanzania-wide screening of non-human primates for infection with the pathogenic bacterium *Treponema pallidum* (Chuma et al., 2018). Details about sample collection, including an ethics statement, and DNA extraction are provided in Chuma et al. (2018). We amplified the complete mitochondrial cytochrome b gene (cyt b , 1140 bp) with newly designed primers 5'-ATGATATGAAAAACCACCGTTG-3' and 5'-CATTTCTAGTTTACAAGGCTAG-3' via PCR. The PCR with a total volume of 30 µ L contained 3 µ L buffer (10 × with 20 mM MgCl ${}_{2}$ ), 0.6 µ L dNTPs (10 nM each), 1 µ L of each primer (1 µ M), 0.2 µ L (1 U) FastStart HighFidelity Taq DNA polymerase (Roche), and 50 ng genomic DNA. Amplification was performed in a laboratory cycler (SensoQuest) with a pre-denaturation step at 95 ${}^{\circ }$ C for 2 min, followed by 40 cycles with 95 ${}^{\circ }$ C for 30 s, 58 ${}^{\circ }$ C for 1 min, and 72 ${}^{\circ }$ C for 1.5 min, and terminated with a final extension step at 72 ${}^{\circ }$ C for 5 min. PCR products were size-separated on 1 % agarose gels, excised from the gel, purified with the QIAquick Gel Extraction Kit (Qiagen), and sent to Eurofins Genomics for Sanger sequencing using both amplification primers. Sequence electropherograms were manually checked and corrected with 4Peaks (https://nucleobytes.com/4peaks/, last access: 6 January 2022​​​​​​​). Newly generated haplotypes were submitted to GenBank and are available under accession numbers OM373544–OM373547.

### 2.2 Phylogenetic analysis

For phylogenetic analyses, haplotypes were aligned with orthologous sequences of another 144 Cercopithecini individuals available in GenBank (Table S2). These mainly derived from museum specimens or wild monkeys (Guschanski et al., 2013; Haus et al., 2013a). The alignment, comprising 148 haplotypes, was conducted with Muscle 3.8.31 (Edgar, 2010) in AliView 1.18 (Larsson, 2014). We reconstructed maximum-likelihood (ML) and Bayesian inference (BI) trees with IQ-TREE 1.6.1 (Nguyen et al., 2015) and MrBayes 3.2.6 (Ronquist et al., 2012), respectively. The ML tree was reconstructed with the best-fit model (TN + F + I + G4) as calculated with ModelFinder (Kalyaanamoorthy et al., 2017) in IQ-TREE under the Bayesian information criterion. Node support was obtained via 10 000 ultra-fast bootstrap (BS) replicates (Hoang et al., 2018). The BI tree in MrBayes was reconstructed via two Markov chain Monte Carlo (MCMC) runs, each with 10 million generations, tree and parameter sampling every 5000 generations, and burn-in of 25 %. As a substitution model, we applied the GTR + I + G model. Using the uncorrected potential scale reduction factor (PSRF) (Gelman and Rubin, 1992), calculated in MrBayes, we checked for convergence of all parameters and adequacy of the burn-in. BI posterior probabilities (PPs) and a consensus phylogram with mean branch lengths from the posterior density of the trees were calculated in MrBayes. Obtained phylogenetic trees were visualized and edited with FigTree 1.4.2 (http://tree.bio.ed.ac.uk/software/figtree/, last access: 6 January 2022​​​​​​​).

Divergence times were estimated with BEAST 2.4.8 (Bouckaert et al., 2014) using a relaxed log-normal clock model of lineage variation (Drummond et al., 2006). As a tree prior we used the Yule model. As a substitution model, we applied the best-fit model as obtained from ModelFinder. As reliable fossil data are unavailable for Cercopithecini internal calibration, we used the age of the most recent common ancestor (MRCA) of Cercopithecini at 9.6 (7.5–11.7) million years ago (Ma) as calculated by Guschanski et al. (2013) based on mitochondrial sequence data. We constrained this node with a normal distribution (mean 9.6, σ 1.05, offset 0.0). This translated into a mean of 9.6 Ma and a 95 % highest posterior density (HPD) interval of 7.5–11.7 Ma. Two independent analyses were run for 100 million generations with sampling every 5000 generations. We used Tracer 1.7.2 (http://beast.bio.ed.ac.uk/Tracer, last access: 6 January 2022​​​​​​​) to inspect the adequacy of a 25 % burn-in and to check for convergence of parameters. We combined sampling distributions of both runs with LogCombiner 2.4.8 and summarized trees with TreeAnnotator 2.4.8 (both part of the BEAST package). The consensus tree was finally visualized with FigTree.

## 3 Results

We successfully sequenced the complete mitochondrial cyt b gene from all 11 *C. mitis* from the four localities in Tanzania. Among them, we found four haplotypes with all individuals from the same locality exhibiting the same haplotype. All newly generated haplotypes were correctly translated into amino acid sequences without any premature stop codons, and sequences show a geographic clustering in the phylogenies. The chance, therefore, that we incorrectly amplified nuclear mitochondrial-like sequences (NUMTs), instead of the true mitochondrial sequences, is minimal. The final alignment comprised 148 cyt b haplotype sequences (4 newly generated and 144 from GenBank).

**Figure 2:**
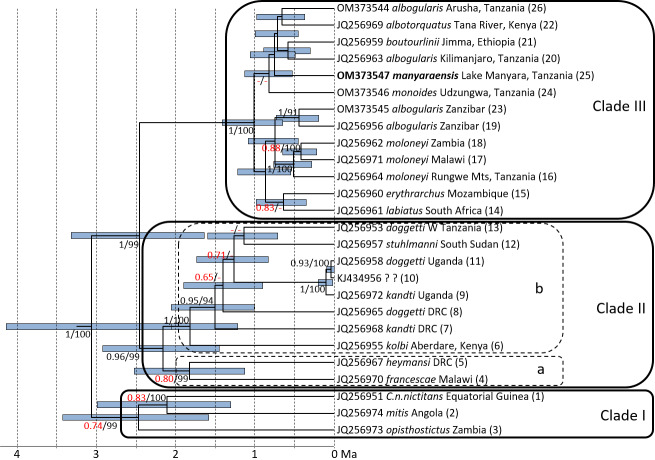
Ultrametric tree based on complete cytochrome b sequences showing phylogenetic relationships and divergence times among mitochondrial lineages of gentle monkeys *Cercopithecus mitis* as inferred from BEAST analysis. Node bars indicate 95 % HPDs. The timescale indicates million of years before present (Ma). Node support (ML BS/Bayesian PP) is given at major nodes. Labels indicate GenBank accession number, subspecies label, sampling location, and haplotype number; I–III indicate main mitochondrial clades (see mitochondrial clades, Fig. 3) (DRC: Democratic Republic of Congo).

**Figure 3:**
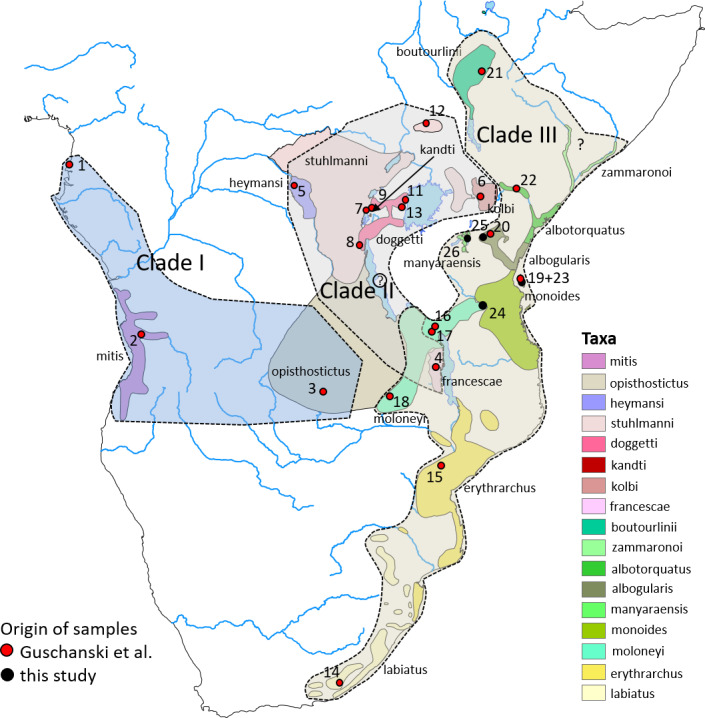
Distribution ranges of gentle monkey *Cercopithecus mitis* taxa and the approximate distribution ranges of the three major mitochondrial clades I–III. Basemap by ESRI.

ML and BI trees revealed almost identical tree topologies with strong node support (ML BS values > 95 %, BI PPs > 0.9) for most major nodes (Figs. 2 and S1–S3). Moreover, the tree topology is largely in agreement with that of previous studies (Guschanski et al., 2013; Haus et al 2013a; Larkin-Gero, 2020). All trees indicate three major mitochondrial clades within *C. mitis*. These do not form monophyletic taxonomic groups but rather reflect a biogeographic pattern. The internal nodes are often not supported (Figs. 2 and 3). Clade I (west-central) includes *C. nictitans nictitans* from Equatorial Guinea, *C. m. mitis* from Angola, and *C. m. opisthostictus* from Zambia. Clade II (central) probably contains two subclades (a and b). Subclade “a” includes *C. m. heymansi* from the Democratic Republic of Congo and *C. m. francescae* from Malawi. Subclade “b” includes *C. m. stuhlmanni* from South Sudan, *C. m. doggetti* and *C. m. kandti* from Uganda, and *C. m. kolbi* from Kenya. Clade III (east) includes populations from Ethiopia southwards into South Africa: *C. m. boutourlinii* from Ethiopia, *C. m. albotorquatus* from Kenya, *C. m. albogularis* from Tanzania (including Zanzibar), *C. m. manyaraensis* and *C. m. monoides* from Tanzania, *C. m. moloneyi* from Tanzania, Malawi, and Zambia, *C. m. erythrarchus* from Mozambique, and *C. m. labiatus* from South Africa.

Clade I diverged from clades II and III 3.06 (4.13–2.05) Ma, and clades II and III split 2.45 (3.32–1.64) Ma. The lineages within Clade I diverged between 2.47 (3.43–1.58) and 2.11 (2.99–1.31) Ma. The divergence times within Clade II are slightly younger: between 2.16 (2.92–1.45) and 1.14 (1.60–0.71) Ma. The most recent divergence times are in Clade III: between 1.01 (1.41–0.65) and 0.42 (0.65–0.22) Ma. The *C. m. manyaraensis* lineage split from its sister lineages *C. m. albogularis* (from Arusha and Kilimanjaro), *C. m. albotorquatus*, and *C. m. boutourlinii* 0.75 (1.06–0.45) Ma.

## 4 Discussion

### 4.1 Phylogeny

*Cercopithecus m. manyaraensis* clusters within the youngest of the three main clades of *C. mitis* taxa. Among its closest relatives are the geographically neighbouring population of *C. m. albogularis* from Arusha and Kilimanjaro. Mitochondrial information alone does not, however, support the subspecific status of *C. m. manyaraensis*.

In general, the topology and the estimated divergence times of our phylogenetic tree are congruent with the results of the study by Guschanski et al. (2013). We, however, used only a short part of the mitochondrial genome (cyt b ) in contrast to Guschanski et al. (2013), who used complete mitochondrial genome sequences. Our study provides some molecular support for the long-standing belief that *C. nictitans* and *C. mitis* are closely related. These two species are similar in many ways, including their vocalizations, ecology, and overall natural history (Kingdon, 2013; Lawes et al., 2013). The haplotype of *C. n. nictitans* from Equatorial Guinea clusters within Clade I together with the central western subspecies *C. m. mitis* and *C. m. opisthostictus*. Assuming that the sampling location and taxonomic classification of the museum samples are correct, this finding supports a close phylogenetic relationship between *C. nictitans* and *C. mitis*.

Similar to Guschanski et al. (2013), our study reveals numerous incongruences between phenotype-based taxonomy, the phylogeny of mitochondrial lineages, and biogeography. Most of the younger divergences within the three clades are statistically not well supported. In Clade II we find paraphyletic relationships among *C. m. kandti*, *C. m. doggetti*, and *C. m. stuhlmanni* (subclade b) and a biogeographically interesting sister relationship between *C. m. heymansi* and *C. m. francescae* (subclade a). These two taxa are separated by a large geographic distance of about 800 km, an area where three other *C. mitis* subspecies occur (*C. m. stuhlmanni*, *C. m. opisthostictus*, and *C. m. moloneyi*). Also interesting is the position of *C. m. kolbi*. Although geographically almost parapatric with a member of Clade III (*C. m. albotorquatus*), it seems to represents the oldest subspecies in Clade IIb and the only representative of this clade east of the Eastern (Gregory) Rift Valley.

In Clade III we find the two southern subspecies clustering together (*C. m. labiatus* and *C. m. erythrarchus*), as well as with *C. m. albogularis* on Zanzibar. *Cercopithecus m. albogularis* from Kilimanjaro and Arusha, however, do not cluster with *C. m. albogularis* from Zanzibar but are more closely related to *C. m. boutourlinii*, *C. m. monoides*, *C. m. albotorquatus*, and *C. m. manyaraensis*. Clade III seems to be the youngest radiation of *C. mitis*, starting approximately 1 Ma.

### 4.2 Phylogeography

A complicated evolutionary history and pattern of para- and polyphyly is also reported for other genera of African primates, such as *Papio* and *Chlorocebus* (Zinner et al., 2009, 2015; Haus et al., 2013a, b; Dolotovskaya et al., 2017). Recurrent expansion and retraction of their ranges, with phases of independent evolution of local populations and phases of gene flow among reconnected populations in response to Pleistocene climate changes, have been proposed to produce such patterns. Incomplete lineage sorting is another possible mechanism that contributed to the paraphyly observed in the mitochondrial tree of *C. mitis*. Analyses of the nuclear genome will likely help identify the mechanisms responsible for the paraphyletic relationships and assess the contribution of gene flow. Even in cases such as *Papio* or *Chlorocebus*, however, where the mitochondrial phylogeny is incongruent with a phenotype-based taxonomy, the distribution of mitochondrial haplotypes provides a geographic pattern that allows some inferences about the evolutionary history of the taxa (Avise, 2009; Zinner et al., 2011; Dolotovskaya et al., 2017).

Since our data comprise only one or a few specimens from each subspecies and location, a phylogeographic scenario has to remain rather speculative. Given the close relationship of the central west *C. mitis* with *C. n. nictitans*, which also represent the first diverging lineages, the geographic distribution of the *C. mitis* haplotypes suggests a west to east range expansion. This expansion might have comprised several waves of colonization and/or different expansion paths north and south of the Western (Albertine) Rift Valley (Guschanski et al., 2013; Butynski and de Jong, 2020; Larkin-Gero, 2020). Ancestors of subclade IIa might have been involved in a first east and south expansion. Notably, *C. m. heymansi* and *C. m. francescae* appear to be relict subspecies. In parallel, members of subclade IIb moved east and north around the Western Rift and, eventually, across the Eastern Rift to the Indian Ocean coast. It has been suggested that *C. m. kolbi* represents a remnant population from this eastward range expansion and retains the ancestral mitochondrial lineage (Guschanski et al., 2013). Members of subclade IIb also expanded their range to the southeast and may have overlapped here with members of subclade IIa. Here it would be interesting to analyse genome data to search for indications of ancient hybridization.

A third range expansion within east and south-east Africa likely began 1 Ma. This probably produced several populations from Ethiopia in the north to eastern South Africa. These populations eventually diverged into the various mitochondrial lineages of Clade III.

### 4.3 Taxonomy

As in *Papio* and *Chlorocebus*, mitochondrial sequence data do not contribute much towards delimiting taxonomic entities within *Cercopithecus*. These data do not support species status for *C. doggetti* and *C. kandti* (Groves, 2001) or subspecies status for any *C. mitis* taxa, including *C. m. manyaraensis*. This, however, does not mean that the taxonomy based on morphology is wrong. As mentioned above, in the case of *C. mitis*, mitochondrial data can help to elucidate parts of the evolutionary history of the taxon and to generate biogeographic insights and hypotheses.

### 4.4 Conclusion

The mitochondrial phylogeny of the *Cercopithecus mitis* complex is only partly statistically supported and shows several paraphylies that are most likely caused by gene flow among taxa and/or by incomplete lineage sorting. The topology of the phylogenetic tree consists of three major clades, with the haplotype of *C. m. manyaraensis* belonging to the youngest clade. *Cercopithecus m. manyaraensis* clusters with the haplotypes of *C. m. boutourlinii*, *C. m. albotorquatus*, *C. m. albogularis*, and *C. m. monoides*. As in other African cercopithecines, such as *Papio* and *Chlorocebus*, the mitochondrial phylogeny is, in part, incongruent with the morphology-based taxonomy and better reflects a geographic pattern.

## Supplement

10.5194/pb-9-11-2022-supplement The supplement related to this article is available online at: https://doi.org/10.5194/pb-9-11-2022-supplement.

## Data Availability

The newly generated sequence data are available at NCBI GenBank; accession numbers: OM373544–OM373547.
