# Supplementary material for: Mito-phylogenetic relationship of the new subspecies of gentle monkey Cercopithecus mitis manyaraensis, Butynski & De Jong, 2020
Source: Primate Biol. 2022 Jun 29;9(1):11–8. doi: 10.5194/pb-9-11-2022 (PMC9285482; doi:10.5194/pb-9-11-2022)
Supplement: The supplement related to this article is available online at: https://doi.org/10.5194/pb-9-11-2022-supplement. [file pb-9-11-supplement.zip › Zinner_supplement_figures.pdf]

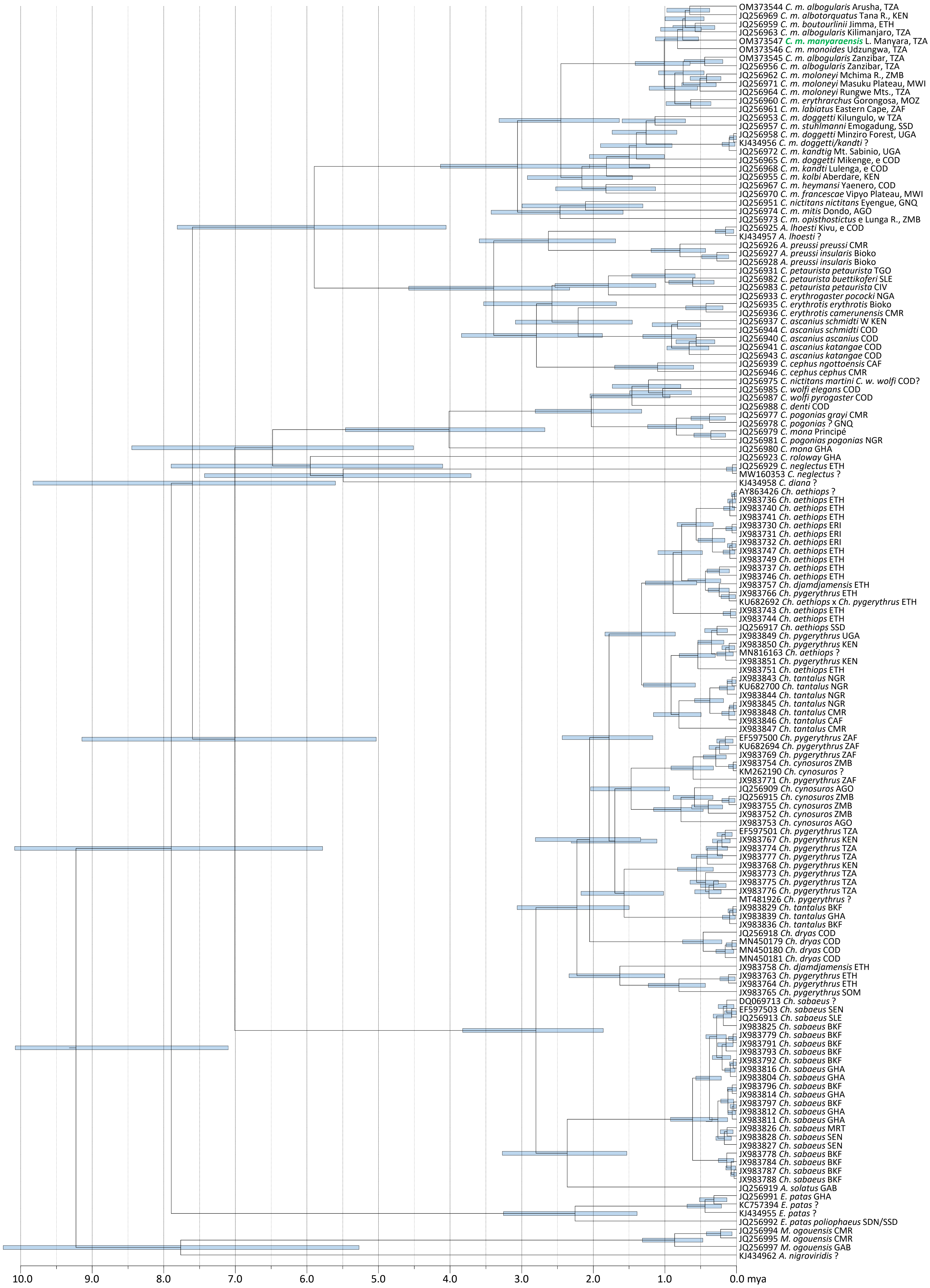

Fig. S1: Ultrametric tree showing phylogenetic relationships among Cercopithecinae based on mitochondrial cytochrome b gene sequences. Given are Genbank accession number, species and geographic origin (countries as three-letter code). The newly described subspecies *Cercopithecus mitis manyarensis* is highlighted in green. Blue bars indicate 95% highest posterior densities. (AGO = Angola; BKF = Burkina Faso; CAF = Central African Republic; CIV = Ivory Coast; CMR = Cameroon; COD = Democratic Republic of Congo; ERI = Eritrea; ETH = Ethiopia; GAB = Gabon; GHA = Ghana; GNQ = Equatorial Guinea; KEN = Kenya; MOZ = Mozambique; MRT = Mauretania; MWI = Malawi; NGR = Nigeria; SDN = Sudan; SEN = Senegal; SLE = Sierra Leone; SOM = Somalia; SSD = South Sudan; TGO = Togo; TZA = Tanzania; UGA = Uganda; ZAF = South Africa; ZMB = Zambia)

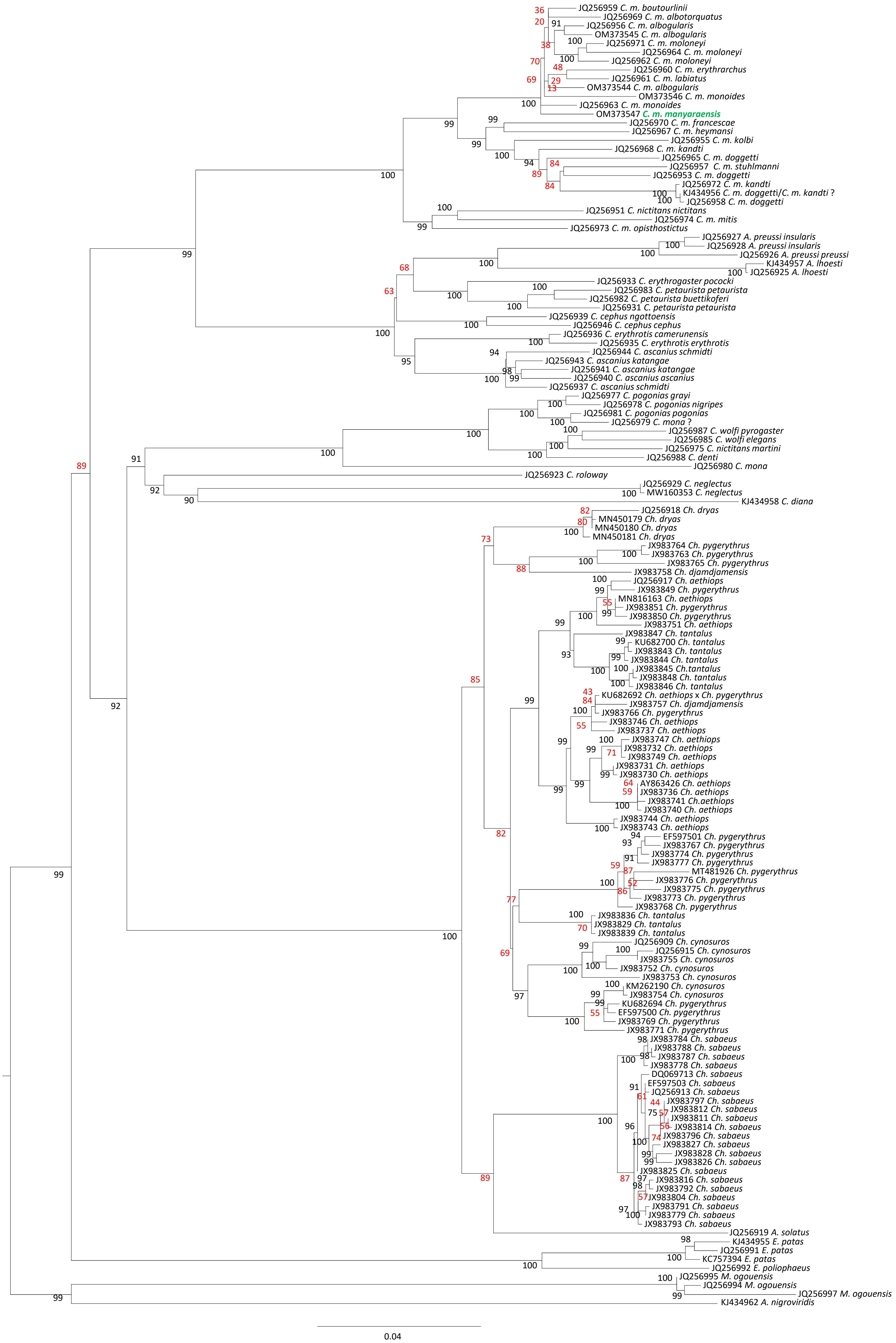

Fig. S2: Maximum-likelihood phylogram showing phylogenetic relationships among Cercopithecinae based on mitochondrial cytochrome b gene sequences. Given are Genbank accession number and species. The newly described subspecies *Cercopithecus mitis manyarensis* is highlighted in green. Numbers at nodes refer to bootstrap values with those <90% indicated in red. The bar below indicates substitutions per site.

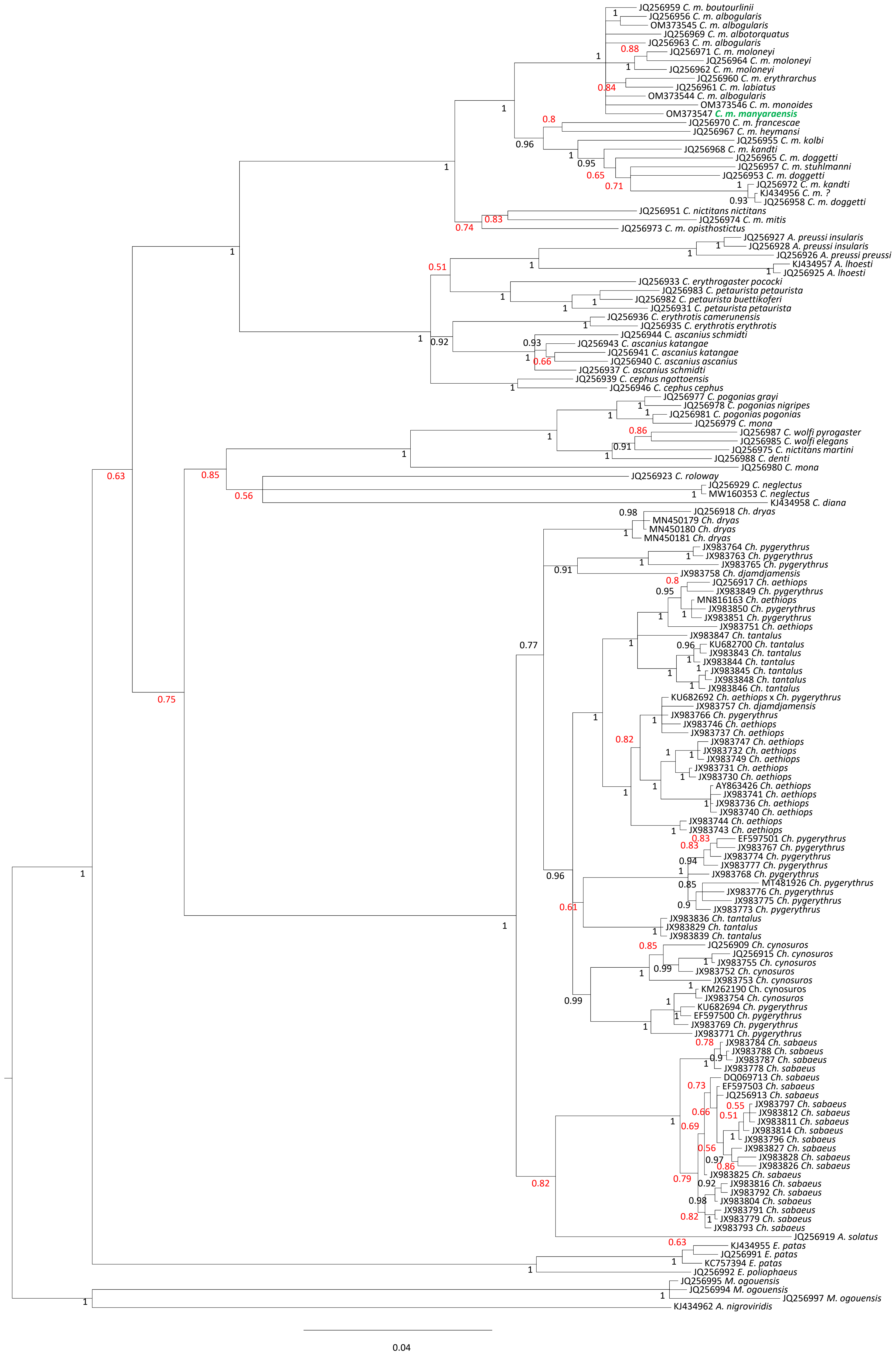

Fig. S3: Bayesian phylogram showing phylogenetic relationships among Cercopithecinae based on mitochondrial cytochrome b gene sequences. Given are Genbank accession number and species. The newly described subspecies *Cercopithecus mitis manyarensis* is highlighted in green. Numbers at nodes refer to posterior probabilities with those <0.90 indicated in red. The bar below indicates substitutions per site.
