# Supplementary material for: Mito-phylogenetic relationship of the new subspecies of gentle monkey Cercopithecus mitis manyaraensis, Butynski & De Jong, 2020
Source: Primate Biol. 2022 Jun 29;9(1):11–8. doi: 10.5194/pb-9-11-2022 (PMC9285482; doi:10.5194/pb-9-11-2022)
Supplement: The supplement related to this article is available online at: https://doi.org/10.5194/pb-9-11-2022-supplement. [file pb-9-11-supplement.zip › pb-9-11-2022-supplement-title-page.pdf]

Supplement of Primate Biol., 9, 11–18, 2022
https://doi.org/10.5194/pb-9-11-2022-supplement
© Author(s) 2022. CC BY 4.0 License.

Supplement of

Mito-phylogenetic relationship of the new subspecies of gentle monkey *Cercopithecus mitis manyaraensis*, Butynski & De Jong, 2020

Dietmar Zinner et al.

Correspondence to: Dietmar Zinner (dzinner@gwdg.de, dzinner@dpz.eu)

- pb-9-11-2022-supplement-title-page.pdf
- Zinner_supplement_figures.pdf
- Zinner_supplement_tables.xlsx

The copyright of individual parts of the supplement might differ from the article licence.
